# Obesity and severe coronavirus disease 2019: molecular mechanisms, paths forward, and therapeutic opportunities

**DOI:** 10.7150/thno.59293

**Published:** 2021-07-13

**Authors:** Tiantian Yan, Rong Xiao, Nannan Wang, Ruoyu Shang, Guoan Lin

**Affiliations:** 1 Military Burn Center, the 990th Hospital of People's Liberation Army Joint Logistics Support Force, Zhumadian, Henan, China; 2 State Key Laboratory of Trauma, Burns, and Combined Injury, Institute of Burn Research, the First Affiliated Hospital of Army Medical University (the Third Military Medical University), Chongqing Key Laboratory for Disease Proteomics, Chongqing, China

**Keywords:** Obesity, Coronavirus disease 2019, Metabolism, Immunity, Inflammation, Thrombosis

## Abstract

Severe acute respiratory syndrome coronavirus 2 (SARS-CoV-2) appears to have higher pathogenicity among patients with obesity. Obesity, termed as body mass index greater than 30 kg/m^2^, has now been demonstrated to be important comorbidity for disease severity during coronavirus disease 2019 (COVID-19) pandemic and associated with adverse events. Unraveling mechanisms behind this phenomenon can assist scientists, clinicians, and policymakers in responding appropriately to the COVID-19 pandemic. In this review, we systemically delineated the potential mechanistic links between obesity and worsening COVID-19 from altered physiology, underlying diseases, metabolism, immunity, cytokine storm, and thrombosis. Problematic ventilation caused by obesity and preexisting medical disorders exacerbate organ dysfunction for patients with obesity. Chronic metabolic disorders, including dyslipidemia, hyperglycemia, vitamin D deficiency, and polymorphisms of metabolism-related genes in obesity, probably aid SARS-CoV-2 intrusion and impair antiviral responses. Obesity-induced inadequate antiviral immunity (interferon, natural killer cells, invariant natural killer T cell, dendritic cell, T cells, B cell) at the early stage of SARS-CoV-2 infection leads to delayed viral elimination, increased viral load, and expedited viral mutation. Cytokine storm, with the defective antiviral immunity, probably contributes to tissue damage and pathological progression, resulting in severe symptoms and poor prognosis. The prothrombotic state, driven in large part by endothelial dysfunction, platelet hyperactivation, hypercoagulability, and impaired fibrinolysis in obesity, also increases the risk of severe COVID-19. These mechanisms in the susceptibility to severe condition also open the possibility for host-directed therapies in population with obesity. By bridging work done in these fields, researchers can gain a holistic view of the paths forward and therapeutic opportunities to break the vicious cycle of obesity and its devastating complications in the next emerging pandemic.

## Introduction

The coronavirus disease 2019 (COVID-19) pandemic continues deteriorating, especially in the winter months before the effects of vaccination become perceptible. As of June 18, 2021, World Health Organization (WHO) had announced more than 177.11 million confirmed cases, including 3.84 million deaths, posing substantial threats to human life and worldwide health systems 1. The clinical manifestations of COVID-19 range from asymptomatic infection to critical illness, and outcomes vary widely from recovery to intensive care unit (ICU) admission and death. Special attention should be paid to critically ill patients due to the high mortality rate. An in-depth understanding of risk factors and molecular mechanisms underlying COVID-19 severity and therapeutic choices can be therefore invaluable in reducing mortality and saving lives.

Obesity, termed as body mass index (BMI) greater than 30 kg/m^2^, is identified as one of the most important independent risk factors for severe COVID-19. Severe acute respiratory syndrome coronavirus 2 (SARS-CoV-2) appeared to be more virulent among patients with obesity. These patients showed a greater tendency to develop adverse events, including respiratory failure, admission to ICU, and invasive mechanical ventilation. In the United States, individuals aged under 60 years old with BMI between 30 and 35 kg/m^2^ and greater than 35 kg/m^2^ were 1.8 and 3.6 times more likely to be admitted to ICU, respectively, compared with patients with BMI less than 30 kg/m^2^ 2. A similar phenomenon was observed in a large prospective analysis of 5279 COVID-19 patients 3. A French study found that BMI greater than 35 kg/m^2^ was associated with a 7.36-fold increase in requiring invasive mechanical ventilation than those with BMI lower than 25 kg/m^2^, even after adjusting for other underlying diseases such as diabetes and hypertension 4. The adverse consequence of obesity was further substantiated by its association with an increased risk of COVID-related death. In China, 88.24% of the deceased had a BMI greater than 25 kg/m^2^, while only 18.95% of the survivors were overweight 5. According to the health analytics platform in England, one of the largest cohort studies to date of clinical factors associated with COVID-19-related death, HR was 1.92 (1.72-2.13) for BMI above 40 kg/m^2^ 6. This phenomenon is reminiscent of the H1N1 pandemic in 2009, in which obesity was also known as important comorbidity for increased disease severity and mortality 7.

Existing evidence from H1N1 and COVID-19 pandemic should serve as a note of caution for modern society. Obesity is such an alarming health problem nowadays that it has been declared as a chronic, progressive, and metabolic disease with low-grade inflammation rather than just a risk factor for other diseases 8. From 1975 to 2014, the proportion of men with obesity worldwide increased from 3.2% to 10.8%, while this data grew from 6.4% to 14.9% in women 9. With a higher expression of the cellular receptor angiotensin-converting enzyme receptor 2 (ACE2) in adipose tissue (AT), people with obesity are more susceptible to SARS-CoV-2 infection 10, 11. Meanwhile, AT can serve as a reservoir after infection and spread SARS-CoV-2 to other organs, just as in influenza A virus, human adenovirus Ad-36, and cytomegalovirus infection 12. Additionally, the health condition of people with obesity is further complicated by the common presence of physiological, metabolic, and immune abnormalities. Coexisting comorbidities such as hypertension, chronic kidney disease, and cardiovascular disease all contribute to severe COVID-19. Taken together, the high prevalence of obesity, susceptibility to SARS-CoV-2 infection, tendency to develop complications, and higher mortality have necessitated the attention to the obese to save lives during the COVID-19 pandemic.

**Figure 1:**
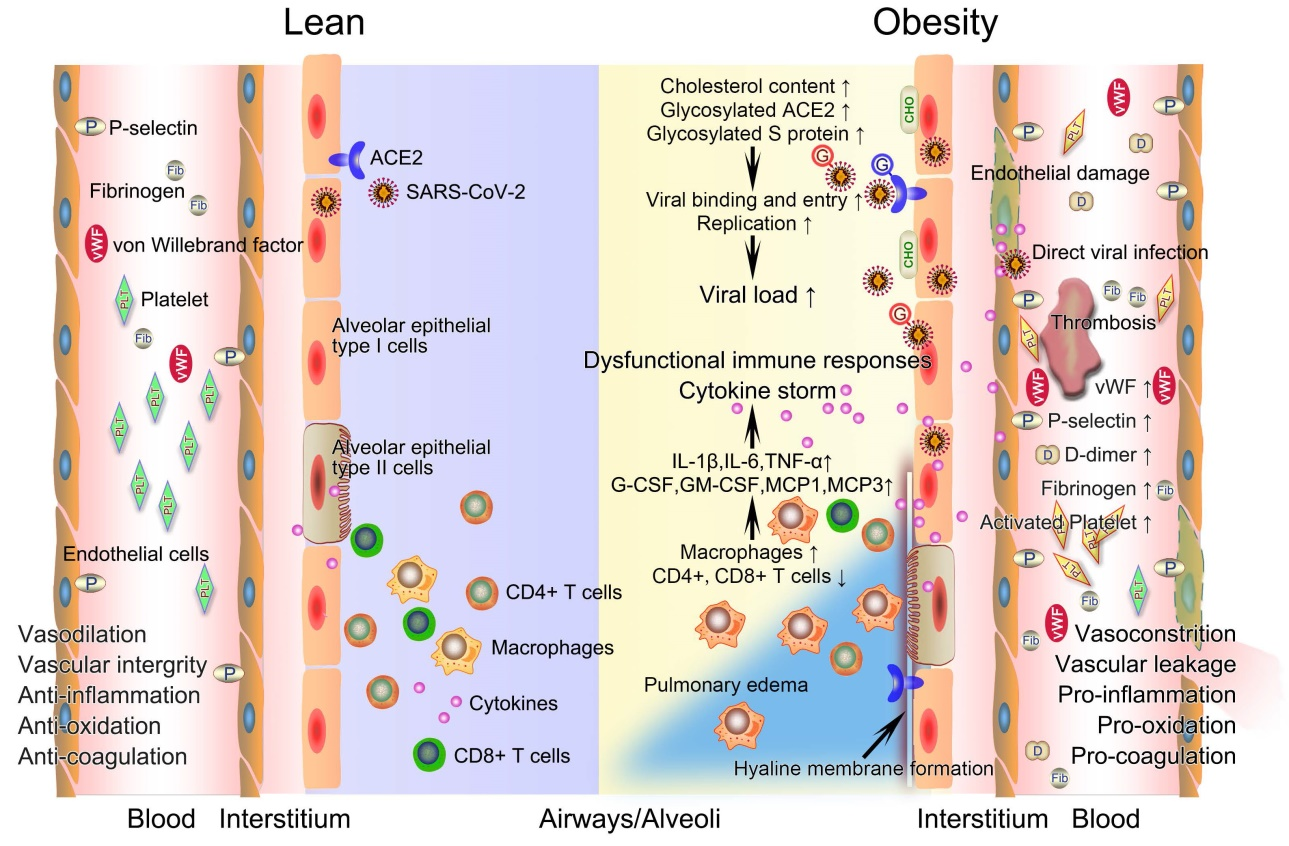
** The potential mechanistic links between obesity and severe COVID-19.** After infection with SARS-CoV-2, innate and acquired immune responses in lean people are effectively activated to clear pathogen and infected cells with minimal inflammation and lung damage. The integrity of their vascular endothelial cells is well-maintained, with vessels possessing normal functions of contraction and dilation, anti-inflammation, anti-oxidation, and anticoagulation. However, obese patients have a higher viral load when exposed to a virus amount equivalent to lean people. Deficiency of anti-viral immunity, runaway of SARS-CoV-2 infections, and excessive infiltration of macrophages jointly contribute to uncontrolled cytokine storm, promoting the development of immunopathology such as pulmonary edema and hyaline membrane. The prothrombotic state of obesity driven in large part by endothelial damage, platelet hyperactivation, hypercoagulability, and impaired fibrinolysis inevitably link obesity with severe COVID-19 by promoting widespread thrombosis. COVID-19, coronavirus disease 2019; CHO, cholesterol; G, glycosylated; SARS-CoV-2, severe acute respiratory syndrome coronavirus 2.

Although obesity has been linked to disease progression in observational or descriptive articles, and the reasons have been briefly discussed in several papers, the mechanisms underlying the COVID-19 pandemic disproportionately affecting patients with obesity remain to be systematically addressed 13, 14. In this review, we expound on the potential mechanisms aggravating disease severity of patients with obesity admitted to hospital for COVID-19 from physiological changes, metabolism, immunity, cytokine storm, and thrombosis, hoping a holistic view and thorough understanding could aid tailored treatments and maximize patient benefit (Figure 1).

## Basic physiological changes induced by obesity increase the susceptibility to severe COVID-19

### Obesity-related respiratory dysfunction

Respiratory dysfunction of individuals with obesity is characterized by low respiratory muscle strength and decreased lung compliance, accompanied by reduced vital capacity, increased residual capacity, and alveolar hypopnea. Excessive AT around the chest and abdomen leads to increased intra-abdominal pressure on the diaphragm, further reducing vital capacity and lung hypoventilation 15. The direct mechanical effect of fat deposition contributes to elevated resistance and premature airway closure, resulting in gas retention, positive end-expiratory pressure, and even respiratory diseases such as sleep apnea syndrome 15. The compensatory respiratory capacity of the obese subject is therefore inadequate and predispose COVID-19 cases with obesity to exacerbated respiratory dysfunction and worsening outcome 16.

### Obesity-related visceral fat deposition

Studies from Italy and Germany, respectively, found that an increase of one unit or 10cm^2^ in visceral fat area measured by CT was associated with an OR of 2.47% (95% CI = 1.02-6.02) or 1.37 (95% CI = 1.07-1.89) for the need of intensive care. The close connection between visceral adipose mass and critical illness was also found in research in China 14. Ectopic fat deposition, especially in abdominal visceral adipose tissue, is common for individuals with obesity. The location of fat distribution determines metabolic risk. In contrast to the metabolic protective effect of gluteofemoral/leg fat, visceral fat obviously worsens insulin resistance, lipid metabolism disorders, and hypertension 17, 18 and also expresses more inflammatory cytokines such as tumor necrosis factor α (TNF-α) and IL-6 19. This secretory profile different from subcutaneous adipose tissue probably contributes to their variations in endocrine function and COVID-19 progression. Even for individuals with normal BMI, increased visceral adipose mass is associated with a greater risk of metabolic disease and mortality. This highlights the limitation of BMI and suggests that the increase in visceral adipose mass is more pernicious in health terms and probably better predict COVID-19 severity than BMI.

### Common presence of other comorbidities

About one in five individuals worldwide have at least one underlying medical condition that puts them at increased risk of severe COVID-19 20. The comorbidities normally presented alone or in combination in the obese include chronic respiratory disease, cardiovascular disease, chronic kidney disease, type 2 diabetes (T2DM), non-alcoholic fatty liver diseases, and metabolic associated fatty liver disease 20, 21. These diseases were identified as independent risk factors for poor COVID-19 prognosis, each contributing to disease progression through different mechanisms 20, 22. For example, physiological abnormalities in patients with NAFLD such as impaired glucose and lipid metabolism, platelet hyperactivation and hypercoagulable state 23, the underlying liver fibrosis 24, up-regulated ACE2 25, and a pronounced inflammatory response 26 probably all contribute to severe COVID-19. In the obese with comorbidities, the mechanisms of comorbidities aggravating COVID-19 and the changes caused by obesity itself synergistically lead to disease progression and severe condition.

## Chronic metabolic disorder of obesity leads to the worsening condition of COVID-19 patients

### Dyslipidemia

Dyslipidemia, a common abnormal state of lipid metabolism in obesity, is characterized by decreased high-density lipoprotein (HDL) cholesterol, increased total cholesterol, triglyceride, and low-density lipoprotein (LDL) cholesterol. Lipid homeostasis is essential for normal lung physiology. For example, HDL acts as the main source of antioxidant vitamin E in type II alveolar epithelial cells 27 and promotes the growth of pulmonary fibroblasts 28 and the production of pulmonary surfactant 29. As a universal and possibly contributory event in chronic lung diseases, dyslipidemia may also be involved in the pathogenesis of severe COVID-19 caused by obesity 30, 31.

High cholesterol in the circulation alters the biophysical properties and function of pulmonary surfactant, leads to cholesterol accumulation in macrophages and other immune cells, and then negatively affects the innate and acquired immune response in the lungs 30, 31. These changes are believed to be critical contributors to the pathogenesis of asthma, pneumonia, acute lung injury, and other lung diseases, as well as increased susceptibility to pulmonary pathogens 30, 31. For diet-induced obesity (DIO), excess fat will be stored in and around normally lean tissues such as the skeletal muscle, liver, and heart. The additional AT leads to an increase of up to 40% of cholesterol loaded into the lung, the most prominent organ affected by COVID-19 32. Moreover, hypercholesterolemia causes circulating cholesterol to be loaded into cells through apolipoprotein E, while chronic inflammation induced by obesity inhibits the unloading process, synergistically increasing cholesterol in cells and forming lipid rafts 33. Cell-loaded cholesterol is essential for the cellular entry of various coronaviruses, such as swine deltacoronavirus and mouse hepatitis virus, and even enhances their pathogenicity in vivo 34, 35. The same rule holds true, in varying degrees, for SARS-CoV 36 and its highly similar analog SARS-CoV-2 32. In the context of COVID-19, high cholesterol facilitates cellular infection of SARS-CoV-2 by augmented lipid raft formation, increasd viral entry sites on the cell surface, and subsequent binding of ACE2 and virus 32. For people with obesity, the high cholesterol in their tissue and cells can increase viral load 32. This finding is validated by studies in which the rapid decreases of total cholesterol and LDL in the blood, defined as hypolipidemia, have been found to be negatively associated with COVID-19 severity and death 37, 38. The reason for hypolipidemia being a predictor of disease progression is that it possibly represents severe loading of cholesterol from the circulation into tissues, hence facilitating SARS-CoV-2 infection and COVID-19 severity.

As a carrier for various bioactive lipid species and lipoxygenase, HDL exhibits significant biological activities, including vasodilation, cellular cholesterol efflux capacity, anti-thrombotic, anti-apoptotic, anti-oxidative, anti-inflammatory, and anti-infectious activities. Besides, HDL also regulates toll-like receptors (TLRs), major histocompatibility complex II, B, and T cell receptors by affecting cholesterol utilization in lipid rafts, serving as a platform for both innate and acquired immunity 39. In the context of dyslipidemic and inflammatory states induced by obesity, HDL showed reduced levels and functional defects characterized by altered composition, abnormal metabolism, and impaired biological activities mentioned above 39. Should people with obesity get infected with SARS-CoV-2, they could experience worsening conditions brought by subnormal and dysfunctional HDL. This hypothesis was given credence by the clinical significance of HDL, whose concentration decreased sharply after infection and changed accordingly with the prognosis in critical COVID-19 patients. The level of serum HDL was positively and negatively correlated with lymphocyte count and C-reactive protein, respectively. Besides, the fluctuations of HDL level were found to be in step with the results acquired from computed tomography examinations and nucleic acid tests, indicating that HDL level was negatively related to COVID-19 severity 40, 41.

The up-regulated expression of lipid metabolism genes in infected pulmonary epithelial cells suggests that SARS-CoV-2, possibly like the H1N1 influenza virus, can target cellular lipid signaling, synthesis, and metabolism to reshape host cells into the optimal environment for viral infection, replication, assembly, secretion, etc. Specific metabolic signatures in serum, liver, lung, and bronchoalveolar lavage fluid (BALF) of H1N1-infected obese mice were discovered, compared with infected lean mice of comparable age 42. The higher levels of phospholipids, cholesterol, and fatty acids in the lungs harvested from obese mice were proved to be closely associated with pulmonary inflammatory overreaction and immune dysfunction, thereby driving more severe pathological changes 42. According to the lessons learned from H1N1, we reasonably infer that dyslipidemia inherent in obesity will be amplified by COVID-19 and lay the foundation for organ damage and increased mortality.

### Hyperglycemia

Obesity-related ectopic fat deposition, especially in the liver, a key insulin-sensitive organ, triggers changes in insulin signaling pathways and insulin resistance. In the inflammatory context of obesity, low-grade chronic inflammation is also present in pancreatic islet, which witnesses the accumulation of mounting immune cells, such as replicating resident macrophages and circulating monocytes. These cells reprogram the immune microenvironment of the pancreatic islet, giving rise to impaired β cell function, islet fibrosis, insulin resistance, and possible hyperglycemia 43, 44. Chronic hyperglycemia can contribute to extensive histopathological changes in lungs, including altered alveolus morphology, thickened alveolar epithelial cells, narrow alveolar space, changed mucus secretion, and increased pulmonary capillary basal lamina and microangiopathy. The consequent decrease in lung function is related to fasting blood glucose index and the duration and severity of glycemic abnormalities 45. Compared with normal people, obese individuals are more prone to deteriorated blood glucose after infected with SARS-CoV-2. Insufficient islet function combined with the direct destructive effect of SARS-CoV-2 on β cells that express ACE2 collectively precipitate acute hyperglycemia 46. Previous studies identified T2DM history and hyperglycemia as risk factors for SARS death and fasting blood glucose as an independent predictor of death 47. Consistently, clinical data demonstrated that abnormally elevated blood glucose (median ≥6.1 mmol/L) independently mediated high risk of progression to critical illness/death from mild/moderate COVID-19 cases as well as increased mortality in critically ill patients 48. It is therefore reasonable that patients with obesity who are more prone to develop hyperglycemia have worse conditions.

Hyperglycemia contributes to severe COVID-19 probably by affecting both virus invasion and body responses. Glucose is not normally present in the thin layer of fluid that covers the epithelium of the respiratory tract, namely airway surface fluid 49. This homeostasis is achieved by the glucose reabsorption of respiratory epithelial cells through sodium-glucose cotransporters. However, hyperglycemia can disrupt this balance and give rise to aberrant glycosylation of proteins, DNA, and lipids in epithelial cells, including ACE2, through directly increasing the glucose concentration in the airway surface fluid 49. The increased glycosylated ACE2 is believed to be responsible for increased binding with SARS-CoV-2 and thereby the severity of COVID-19 infection 50. In addition, the spike (S) protein of SARS-CoV-2 is cleaved into S1 and S2 subunit by protease furin. S1 binds to ACE2, while the highly glycosylated S2 subunit is responsible for the fusion of the SARS-CoV-2-ACE2 complex with the cell membrane and subsequent entry 50. Conceivably, the combination of highly glycosylated S protein and ACE2 brought by hyperglycemia favors the cellular invasion of SARS-CoV2 and following infection of multiple organs. Meanwhile, hyperglycemia also impairs major components of the immune system that copes with infection 51. For instance, high glucose inhibits the production of type I interferon (IFN), leading to decreased antiviral activity at the cellular level 52. Human and animal hyperglycemia-related studies both demonstrated the reduced formation of extracellular traps 53, impaired neutrophil activity (e.g., chemotaxis, phagocytosis) in a protein kinase C-dependent way, and thus failure of pathogen elimination 54. Glycosylation of immune proteins under a hyperglycemic state also poses threats to immune function; for example, glycosylated complement C3 with abnormal tertiary structure inhibits the fixation with pathogens and decreases phagocytosis 55. Therefore, hyperglycemia probably aids SARS-CoV-2 intrusion and impairs body antiviral responses, thereby causing severe symptoms and worse outcomes in obese patients.

### Vitamin D deficiency

Unlike other disputed vitamins in obesity, vitamin D deficiency in people with obesity is highly prevalent and well-documented, as evidenced by the negative impact of high body fat percentage and BMI on serum 25-hydroxyvitamin D (the major circulating vitamin D metabolite) level 56. Multiple comorbidities of obesity, such as impaired pancreatic islet function and cardiac metabolic diseases, are all associated with vitamin D deficiency 57, 58. Physiological effects of vitamin D in the lungs include induction of autophagy, generation of antimicrobial peptides that lower viral replication rate, and synthesis of reactive oxygen intermediates and reactive nitrogen intermediates. Besides, vitamin D regulates the renin-angiotensin system (RAS) and maintains pro- and anti-inflammatory cytokines balance, thereby helping control respiratory tract infection 59, 60. Accordingly, vitamin D deficiency can worsen COVID-19 by impeding the activation of defense pathways above, triggering cytokine storms, and aiding immune dysfunction 59, 61, 62. The effects of inadequate vitamin D in COVID-19 are substantiated by a preliminary study showing a striking correlation between disease severity and the epidemiology of vitamin D deficiency regarding population, climate, and regions 61. That is, it is logical to speculate that obese COVID-19 victims without sufficient vitamin D are predisposed to an increased risk of severe conditions.

### Polymorphisms of metabolism-related genes

The formation of obesity is influenced by, environmental and genetic factors such as diet and physical activities. Individual differences in weight gain and the occurrence of different phenotypes of obesity under a similar obesogenic environment indicate the significance of genetic differences. Currently, over twenty genes have been found to be involved in metabolism and fat deposition, among which melanocortin 4 receptor (MC4R), fat mass and obesity-associated gene (FTO), and peroxisome proliferator-activated receptor γ (PPARγ) have been extensively studied 63. As the most common monogenic cause of obesity, deficient MC4R and the central melanocortin pathway together regulate energy intake, expenditure, and homeostasis 64. FTO single nucleotide polymorphism is established as a key player in obesity and genetic aging by regulating dietary behavior, telomere length, and cell nutrition perception through amino acids 65. In addition to regulating glucose and lipid metabolism, PPARγ is also part of the cytokine loop as an anti-inflammatory player after viral infection, promising to be a therapeutic target for COVID-19 66. Previous researches reported that several individual gene polymorphisms such as interleukin 1 (IL-1), IL-1α, and interferon regulatory factor 9 (IRF9) are associated with disease severity and/or clinical outcome of influenza virus infections 67 and SARS 68. Considering the close connection between metabolic gene polymorphisms and obesity, the possibility that these targets (e.g., MC4R, FTO, PPARγ) in obese patients are more directly related to severe COVID-19 than BMI cannot be excluded. It is worthwhile to keep track of whether and how metabolic gene polymorphisms are related to severe COVID-19, helping pinpoint populations vulnerable to severe infection.

## Dysfunctional antiviral immunity induced by obesity fails to eliminate SARS-CoV-2 and might explain severe COVID-19

**Figure 2:**
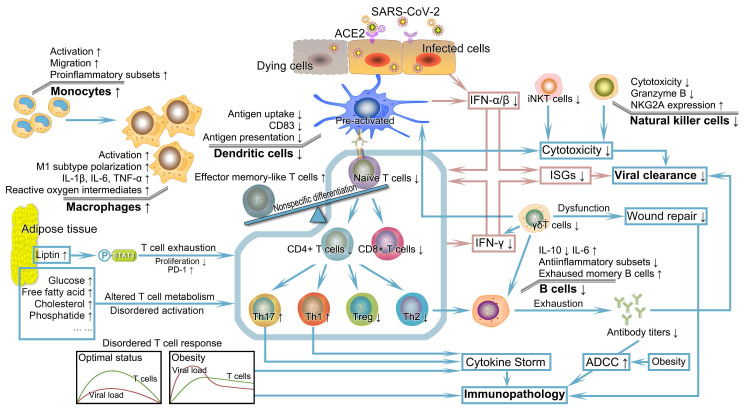
** Severely deficient anti-viral immunity and its subsequent catastrophic effect in patients with obesity encountered SARS-CoV-2.** Patients with obesity probably have significant defects in multiple aspects of innate and acquired immunity. IFN, the first pivotal defense line against the virus, exhibits reduced production, faulted IFN signal transduction, and pathogenic interferon stimulating genes, leading to uncontrolled viral replication and transmission. DCs with numerical and functional defects in obesity demonstrate impaired ability to stimulate naïve T cell expansion. The resulting insufficient number and unbalanced subsets of T cells combining obesity-related thymic degeneration, T cell senescence, impaired initiation, delayed timing of T cell response relative to viral replication, and other adverse factors in the context of obesity fail to elicit a potent immune response. The altered number and function of B cells and decreased antibody titers cannot neutralize and inactivate SARS-CoV-2. Coupled with the abnormalities of other cells such as NK cells, iNK cells, macrophages, and γδT cells, deficient anti-viral immunity caused by obesity probably drives the formation of systemic cytokine storm and progression of immunopathology, possibly followed by dysregulation of tissue repair. DC, dendritic cell; IFN, interferon; iNKT cells, invariant natural killer T cells; NK cells, natural killer cells; SARS-CoV-2, severe acute respiratory syndrome coronavirus 2.

Once infected with SARS-CoV-2, the human body will initiate an antiviral response that depends on the coordination of well-functioning innate and acquired immunity to eliminate pathogens. However, the ectopic fat deposition in immune tissues such as the thymus, spleen, bone marrow, and lymph nodes lead to destructed tissue integrity and an altered cellular environment 69. Consistent with the dependence of defensive function conferred by the immune system on the proper development and maturity of immune cells, the lymphoid tissue microenvironment undermined by obesity gives rise to alterations in the development, phenotype, diversity, and activity of immune cells. These alterations complicate and further perpetuate impaired innate and acquired immunity as well as their interactions, individually or synergistically increasing the risk of worse prognosis in obese patients infected with SARS-CoV-2 (Figure 2) 69-71.

### IFN

IFN, which is classified into three types, is a kind of secretory protein induced by innate immune sensors perceiving pathogen-related molecular patterns through pattern recognition receptors such as retinoic acid-inducible gene I receptors and TLRs. In IFN-I, the most widely expressed and clearly defined is IFN-α and IFN-β, while IFN-II family consists of a single gene product IFN-γ, and IFN-III is a variety of subtypes of IFN-λ 72. The ubiquitously distributed receptors of IFN-I and IFN-II determined that they can act on most of the cells with extensive overlap between their signal pathways. Taking IFN-α as an example, it exerts pleiotropic effects in the combat against infection, including but not limited to enhanced responses of dendritic cells (DCs), natural killer (NK) cells, T cells, and B cells through differential activation of signal transducer and activator of transcription (STAT) molecules. The induction of interferon-stimulated genes (ISGs) by IFN-I largely contributes to restricting the viral replication cycle, while the upregulation of the major histocompatibility complex augments the lysis of infected cells 72. IFN-III receptors are preferentially expressed on the epithelial cells of the respiratory system, gastrointestinal tract, and reproductive system. When combined with the receptor, IFN-III could locally control the viral infection at the entry sites such as the respiratory tract 73. Altogether, IFN constitutes the first line of defense against the virus by establishing antiviral cellular status and activating subsequent immune responses.

In the long struggle for survival, viruses, including SARS-CoV-2, have adopted several strategies against antiviral immunity. Being different from the Middle East respiratory syndrome coronavirus, human parainfluenza virus 3, respiratory syncytial virus, and influenza A virus, SARS-CoV-2 showed an extremely low level of IFN-I and IFN-III along with mild ISG responses in human, animals and cells in vitro 74. The potential mechanism was that viral membrane protein prevented components such as retinoic acid-inducible gene I and mitochondrial antiviral signaling from forming multiprotein complex, resulting in impeded nuclear translocation and activation of IRF3 75. Corresponding to the laboratory evidence, clinical studies found that COVID-19 patients with severe IFN-α deficiency had longer ICU stay, higher viral load, and worse prognosis 76, 77. In addition to viral factors, specific host factors are also involved in determining the outcome of IFN signal transduction. Patients with obesity fail to launch a robust IFN-I response, resulting in the deterioration of H1N1 infection 78, 79. It is conjectured that this destructive IFN response may increase the susceptibility of individuals with obesity to severe COVID-19. Things like reduced IFN production, faulted IFN signaling, pathogenic ISG effectors, and possibly accelerated viral mutation in obesity collectively lead to impaired antiviral ability and extensive cytopathogenic effect induced by uncontrolled SARS-CoV-2 at the early stage of COVID-19.

IFN production may be significantly reduced at first. Compared with the control group, obese mice infected with H1N1 showed a dramatically decreased level of IFN-α and IFN-β in the BALF and culture supernatant of alveolar macrophages. The viral load and mortality were also higher 79. In line with animal experiments, H1N1 patients with obesity showed impaired production of IFN-I, probably related to leptin 80. This reduction could be caused by the imperfect development of immune cells and their shift towards inflammatory phenotypes 70, 71. Coincidentally, the decrease in IFN-γ production in obese people infected with influenza A virus is related to dysfunctional γδΤ cells, whose Τ cell receptor consists of γ and δ chain. The subnormal number of γδT cells, together with the surviving ones being unresponsive to TLR ligands and inactive IFN responses synergistically contribute to IFN-γ deficiency 81.

Moreover, the IFN signal transduction cascade may be inhibited. Typically, the binding of existing IFNs and the corresponding receptors leads to Janus Kinase-STAT being phosphorylated and translocating into the nucleus in a complex with IRF9. This trimeric complex then binds to the interferon-stimulated response elements and causes transcriptional induction of antiviral ISGs. However, chronically increased leptin produced by AT in obesity interferes with the IFN signal transduction through increasing suppressor of cytokine signaling 3, which negatively regulates the Janus Kinase-STAT pathway, causing the limited IFN in vivo to fail in inducing antiviral ISGs transcription 80, 82.

The disordered IFN-I is pathogenic. In addition to expression level, the timing of IFN production is also important. As mentioned above, obesity would cause severe symptoms and adverse prognosis of H1N1 patients because of disordered IFN production and ISG responses 78-80. Previous studies showed that IFN responses were more likely to delay rather than disappear. Instead of being protective against viral infection as supposed, disordered IFN-I response emerged as a critical driver for developing severe lung injury in SARS mice, facilitating massive immune cell infiltration, excessive inflammatory responses, vascular infiltration, and alveolar edema formation 83. The aberrant intensity and duration of ISGs even determined the viral load and disease severity of COVID-19 patients 84. Therefore, based on other coronaviruses' parallel immunological and pathophysiological features, it can be speculated that pathogenic IFN-I response in obesity as shown in H1N1 can also drive the immunopathological changes, COVID-19 progression, and mortality. However, the dynamics and interaction of viral replication and the IFN signaling pathway are still an open question that deserves further attention. Identifying the turning point of IFN function from protective to pathogenic is crucial for determining the therapeutic window.

Finally, ineffective IFN responses facilitate viral mutation. As a typical RNA virus, SARS-CoV-2 shares the characteristic of genetic material being highly mutable. The influence of mutations on viral replication kinetics and infectivity in vitro and clinical significance in vivo has been demonstrated in mounting reports 85. For example, strain B.1.1.7 is a new variant more transmissible and likely to escape from human immunity. Being recently brought to light and became the dominant strain in the London area, strain B.1.1.7 prompted renewed alarm and escalation of control measures 86. As shown in obese mice with H1N1, decreased type I IFN responses were associated with the rapidly emerged viral variants exhibiting increased replication, enhanced pathogenic, and more diverse quasispecies 87, 88. The results showed that obesity-related impaired IFN responses afford the emergence of a more virulent virus population capable of inducing greater disease severity. This phenomenon highlights the need for vigilance in the clinical management of COVID-19 patients with obesity, as the emergence of potentially pathogenic variants is not specific to a particular obesity model or virus strain but a regular pattern 87, 88. The analogy between the H1N1 virus and SARS-CoV-2 makes people wonder whether SARS-CoV-2 mutation, infectivity, and virulence will be influenced by obesity. Thus, further independent studies are needed to confirm or disprove this idea.

### NK cells

NK cells directly kill infected targets by secreting lytic particles containing apoptosis-inducing granzymes and perforin, thus acting as major players in protective immunity against viral infections. It is shown that the increased risk of cancer and infections attributable to numerical and functional defects of NK cells in human and experimental animals with obesity 89-91. The cytotoxic activity of NK cells in obese mice infected with influenza virus was 50% lower than that in the control group, and the NK cell-mediated killing was also naturally reduced 89. This phenomenon is related to the abnormal metabolism of NK cells caused by obesity. Once mobilized, NK cells would show special metabolic flexibility, converting from oxidative phosphorylation to glycolysis to meet the increased energy needs 91, 92. The increased PPARs in obesity drive the inhibition of glycolysis, downregulated production of IFN-γ and cytotoxic granules in NK cells, leading to blunted antiviral activity 90. Lipotoxic obese environment such as the chronic elevation of LDL, triglyceride, and circulating free fatty acids (FFAs), induces a large accumulation of lipid in NK cells, thus interferes with cellular bioenergetics and results in complete “paralysis” of cellular metabolism and transition 90. However, the effect of obesity on NK cells goes far beyond metabolic reprogramming. High levels of IL-6 secreted by AT could downregulate NK cell cytotoxicity by inhibiting the expression of granzyme B and perforin without altered granule exocytosis in both obese human and murine settings 93. In contrast to the central role of leptin in modulating NK cell number and activity in lean individuals, post-receptor signaling components (protein kinase B pT308, Janus kinase-2p) of leptin were differentially abrogated despite the up-regulated leptin and leptin receptors in subjects with obesity 94. This functional desensitization is also responsible for the insufficient function of NK cells in obesity. In addition, the frequency of NKG2A-positive NK cells increased as BMI elevated in patients with new-onset of T2DM 95. Binding to non-classical human leukocyte antigen E, NKG2A suppressed cytotoxicity of NK cells and contributed to the poor control of human immunodeficiency virus 1 infection. Analogously, BMI-related increased NKG2A in obesity may also facilitate the immune escape of SARS-CoV-2 96.

In conclusion, NK cells in people with obesity cannot limit SARS-CoV-2 transmission as expected, nor restrain systemic inflammation by killing the activated inflammatory dendritic cells, monocytes, or T cells 97. Although this hypothesis needs further clarification, it alludes to potential mechanisms for poor outcomes of COVID-19 patients with obesity.

### Invariant natural killer T (iNKT) cell

iNKT cells constitute a distinct T lymphocyte subset co-expressing NK-lineage receptor and an invariant T-cell receptor. After being activated, the iNK T cell, as an immunomodulator, mobilizes other immune cells to participate in the anti-viral chorus by secreting cytokines such as IL-4, IL-10, and IFN-γ. The expression of effector molecules on iNKT cells like perforin and Fas ligand also endows cytotoxic effects 98, 99. Under physiological conditions, iNKT cells were found highly abundant in mice and human AT. However, the number of iNKT cells decreased as AT expanded during the development of obesity and correlated inversely with the infiltration of pro-inflammatory macrophages. This reduction can be restored by weight loss of humans and mice 100. Moreover, the *vitro* studies showed that the cytokine secretion and resultant function of surviving iNKT cells were inhibited by leptin and leptin receptor pathways which also reduced iNKT cell number 101. It is therefore conceivable that once confronted with SARS-CoV-2, iNKT cells with numerical and functional defects in patients with obesity will be unable to link antigen-presenting cells with local or acquired immune cells (NK cells, T cells, and B cells) and are not conducive to virus clearance 98.

### DCs

DCs are critical immune sentinels in response to viral infection. As the most potent and exclusive professional antigen-presenting cells, DCs can trigger naïve T cells and function critically in initiating and maintaining cellular immune responses. The crosstalk between DCs and adipocytes maintains or even dominates the immune homeostasis of AT, indicating the critical role of DCs in AT 102, 103. D O'Shea et al. found that circulating DCs in the obese showed significantly reduced count and functional defects featured by lower expression of CD83 following TLRs stimulation when compared with lean controls. As an essential molecule implicated in the elicitation of T cell responses, the insufficient level of CD83 leads to declined antiviral capacity and an increased likelihood of severe viral infection in patients with obesity 104. Relatively high levels of cytokines such as granulocyte-macrophage colony-stimulating factor and IL-6 induced by obesity can result in the pre-activation of DCs with, however, increased risk of dysfunction and activation-induced apoptosis. Despite the similar phenotype in the control group, DCs in DIO mice showed blunt ability to stimulate naïve T cell expansion because of upregulated serum cytokines and chemokines, including IL-1α, IL-17, and TNF-α 105. Moreover, obesity also impairs the migration of DCs to lymph nodes 106. Therefore, DCs in the obese are not competent to initiate required immune responses to cope with the pathogen, especially lately emerged SARS-CoV-2 that carries novel antigens first encountered by the human body.

Further support for the impaired function of DCs in patients with obesity is suggested by their impaired antigen presentation and inadequate capability of guiding antiviral orchestration of T cells when encountered influenza virus. This defect was closely associated with up-regulated IL-6-driven proinflammatory state in the lungs of DIO mice 107, 108. Similarly, one of the important immune features of severe COVID-19 was the dramatic reduction of the proportion of myeloid and plasmacytoid DC in BALF. In particular, the degree of plasmacytoid DC depletion correlated strongly with disease severity 109, 110. In the meantime, COVID-19 also leads to low expression of maturation markers in DCs, high expression of programmed cell death ligand 1, inability to upregulate DC80 and CD86 in response to ssRNA and other stimuli, decreased secretion of IFN-α and IFN-β, and impaired ability to stimulate T cell proliferation 111, 112. Considering the weakening of antiviral responses and viral clearance by altered DCs in people with obesity, the close causality between the development of severe COVID-19 and abnormal DCs is therefore reasonable.

### T cells

As the indispensable components of the immune system, T cells harmonize and maintain many aspects of anti-viral responses. Recently, Cañete et al. have highlighted the importance of T cells in response to SARS-CoV-2 by showing that virus-specific memory T cells might achieve immunologic memory despite the blunt long-lived antibody responses in COVID-19 113. Hence, impaired antiviral responses and deteriorated COVID-19 may occur in the setting of detrimentally altered development, number, and function of T cells consequent to obesity.

Immune senescence, commonly seen in the elderly, is accelerated by obesity as it promotes thymic degeneration and T cell senescence 71, 114. This aging immunity was even found in children with obesity 115. T lymphocyte DNA hypermethylation in humans and animals with obesity was epigenetic evidence supporting obesity-related T cell senescence 116, 117. Article published by Williamson et al., one of the largest cohort studies on clinical factors associated with COVID-19-related death so far, showed that aging is strongly associated with the risk of COVID-19-related death 6. The risk for patients aged ≥80 years old increased more than 20-fold than for those aged 50-59 years old 6. By analogy with the significant contribution of age-related immunosenescence to the high mortality rate of elderly patients, it is reasonably speculated that similar deficient immunity in the COVID-19 patients with obesity may also confer susceptibility to disease exacerbation and fatal outcomes 118.

As mentioned above, malfunctioning DCs from DIO mice result in impaired initiation of specific T cell responses 105. In high-fat diet-fed animals and humans, CD4+ T cells preferentially differentiated toward into a type of effector memory-like T cell which was independent of the crosstalk between DCs and T cells 119. This non-specific differentiation depletes the number of naïve and memory T cells, leading to decreased proportion of specific T cells that respond effectively to foreign antigens. Multi-omics studies of patients suffering from COVID-19 demonstrated that CD4+ naïve T cells could also differentiate into a group of clonally expanded nonspecific CD4+ cytotoxic phenotype 120. Insufficient initiation and nonspecific depletion combine to render T cells unable to mobilize rapidly and effectively when confronted with novel antigens like SARS-CoV-2, leading to insufficient supply of effector and helper T cells owning specific antiviral functions. In addition, previous data showed that DIO could lead to a 44% decrease in CD8+ T cells 121, while controversial studies claimed that the number and frequency of influenza virus-specific CD8+ T cells in the lung, rather than the total population, were affected by obesity 108. One of the prominent immunological features among severe COVID-19 patients was lymphopenia with decreased CD4+, CD8+ T, and Treg cells. Obesity and lymphopenia, especially preferential decline in CD8+ T cells, can serve as predictors of poor prognosis in COVID-19 patients 122. The decrease in T cells arising from coronavirus infection and obesity together conduce to a higher possibility of critical condition in patients with obesity. However, the reciprocal interplay between obesity and disease progression may not be unilateral. On the one hand, the immune defenses of patients with obesity are inherently weakened because of immune senescence and inadequate T cells. On the other hand, the declined naïve T cell response is crucial in an exuberant inflammatory response and cytokine storm 123. It is worth keeping track of whether the number or frequency of CD4+ and/or CD8+ T cells in peripheral blood and pulmonary inflammatory tissue decrease in obese patients with severe COVID-19. More efforts are warranted to ascertain the mechanistic role of these alterations in disease pathogenesis and progression.

Functional defect of T cells across multiple species with obesity is also a critical point to be reckoned. Compared with nonobese controls, both human and animal models with obesity consistently revealed attenuated proliferative capacity and increased exhaustion of T cells, as indicated by down-regulated ki67 and up-regulated PD-1, respectively. This is further corroborated by the reduced IFN-γ and TNF-α production in stimulated polyclonal T cells from subjects with obesity 124. These dysfunctions could increase cell apoptosis and prevent T cells from proliferating and activating effectively when exposed to acute viral infections. The chances of obese COVID-19 patients with obesity becoming seriously ill are bound to be significantly increased in the absence of effective antiviral T cell response. These concerns were substantiated by the runaway influenza virus and worsening illness experienced by obese individuals with influenza 125. Viruses such as SARS-CoV-2 can negatively affect the T cell response. Two unusual subtypes of CD4+ T cells called clonally expanded cytotoxic phenotype, and proliferative exhausted phenotype were associated with disease severity in multi-omics studies, while the polyfunctionality and cytotoxicity of CD8+ T cells were attenuated in severe COVID-19 patients 120. It thus logically follows that malfunctional T cells caused by a combination of host and viral factors predispose COVID-19 patients with obesity to the failure of virus control and more severe conditions. The reasons for T cell dysfunction in patients with obesity are various. First of all, high leptin levels led to the upregulation of phosphorylated STAT3, which interacted with the PD-1 gene promoter and induced its expression on T cells, leading to cell exhaustion 124. Then T cells switch the balance of the metabolic program from catabolism in the quiescent state to anabolism in the activated state to meet the energy required by the effective immune response to kill pathogens. Compared with ordinary people, extra increases in glucose, FFAs, cholesterol, phospholipids, and other metabolites in people with obesity mechanically alter the metabolism of T cells, leading to activation disorder and declined activity 42, 119, 126. Finally, T cell dysfunction in people with obesity is also partially driven by IFNs deficiency at the early stage of infection. Instead of being protective and stimulative in T cell response, subsequent delayed IFNs inhibit T cell proliferation, prevent its outflow from lymphatic organs, and cause T cell exhaustion and death 127.

The T cell immunity in response to SARS-CoV-2 can act as a double-edged sword being antiviral effective or driving immunopathological development 128, 129. The choice of being “friend” or “foe” depends on its timing of initiation, duration, and level, possibly. T cell response was found to peak around one to two weeks after SARS-CoV-2 infection while the patient was turning to the early clinical stage from the onset of symptom 130. For patients with obesity, further delay and incompetence of antiviral T cell response in the early stage of COVID-19 would happen because of the impaired initiation of naïve T cells after receiving antigen presentation, decreased proliferation, and impaired function of T cells. These T cells fail to control highly replicating and possibly mutating viruses, and tend to fuel cytokine storms instead. In addition, obesity can also lead to altered cell subsets, with increased pro-inflammatory Th1 and Th17 cells and decreased anti-inflammatory Th2 and Treg cells, contributing to excessive inflammatory reaction 131. The occurrence of progression from mild to severe cases of COVID-19 mainly in the second week from the onset of symptoms coincided with the obesity-delayed T cell response. Researches revealed the heightened chemokines (CXCL8, CXCL9, CXCL10) induced by circulating IFN and activated cytolytic Th1 cells phenotype in severe COVID-19 patients, yet their virus-specific responses were similar to that in mild patients 132.

Epithelial γδ T cells are worthy of attention because of their unique connection with epithelial tissue. Except for the protective antiviral function in influenza pneumonia, γδ T cells can provide growth factors, recruit macrophages, and neutrophils during wound repair. γδ T cells also can induce DC maturation, interact with Tregs, and participate in the restoration of epithelial barrier 133. However, in COVID-19 patients with obesity, γδ T cells are negatively subjected to the combination of viral and host factors possibly. A decreased number of γδ T cells were found in COVID-19 cases 134. In the inflammatory context induced by obesity, γδ T cells showed reduced number, impaired homeostasis and antiviral function in humans due to their high sensitivity to inflammation, leading to poor repair of lung lesions and worse pathological changes 81. In unraveling the mysteries in COVID-19 victims with obesity, more data are needed to ascertain the mechanistic role of altered γδ T cell responses from the overlay of obesity and SARS-CoV-2 in immunopathology and disease progression.

### B cell

Obese individuals infected with the H1N1 influenza virus showed lower levels of specific antibodies and weaker neutralization ability 135, 136. This can be ascribed to various reasons, such as the inflammatory environment in vivo, DNA hypermethylation of B cells, and abnormal leptin that fails to regulate the development, maturation, and activity of B cells 116, 117. The peripheral B cell pool in subjects with obesity is characterized by the increased proportion of pro-inflammatory late/exhausted memory B subsets and decreased anti-inflammatory transitional B cells 137. In addition to the reduced number and imbalanced subgroups, functional defects of B cells in individuals with obesity possibly participate in the formation of cytokine storm by secreting more pro-inflammatory cytokines (e.g., IL-6). It is rational that obesity leads to severe COVID-19 by altering the number and function of B cells and their interaction with other lymphocytes (such as T follicular helper cells) through the inflammatory environment and adipokine imbalance.

SARS-CoV-2 infection elicits a potent humoral immunity critical for the clearance of cytopathic viruses which is proved by rapid and near-universal detection of virus-specific antibodies in the several days following infection 138. There is little information regarding host response and viral dynamics following SARS-CoV-2 infection in populations with obesity. The higher risk of more severe clinical prognosis with higher titers of antibodies suggests that robust antibody response alone is insufficient to avoid severe illness 139. Considering that antibody-dependent cellular cytotoxicity probably aggravates cytokine storm and disease conditions in mice with obesity, it is necessary to find out whether a similar situation is present in obese patients with COVID-19 140.

## Obesity creates a conducive ground for the cytokine storm in COVID-19 cases

SARS-CoV-2 can elicit an exuberant cytokine storm both in vitro and in critically ill patients, a pathological state in which cytokines are rapidly released to form a hyperinflammation cascade. The occurrence of immunopathology, multiorgan failure, and even death in COVID-19 are at least partially to be blamed on cytokine storm 141, 142. This finding parallels the situation described in SARS 143 and H1N1 144, in which similar dysregulation drives the tissue damage and pathological progression. Data about the cytokines in COVID-19 patients with obesity are currently scarce. However, previous studies on H1N1 influenza have provided noteworthy findings. Compared with lean mice, the expressions of inflammatory cytokines such as IL-6, TNF-α, and IL-1β and chemokines such as monocyte chemotactic protein 1 in the lungs of mice with obesity were delayed and decreased in the early stage after infection 79, 89, 145. However, excess cytokine secretion then occurs and resulted in cytokine storms in mice with obesity 42, 89, 145. Similarly, obesity also predisposes patients infected with SARS-CoV-2 to systemic cytokine storm, possibly, owing to different but synergetic mechanisms.

1) Low-grade inflammation in obesity. Obesity is a chronic low-grade inflammatory disease featured by elevated levels of inflammatory cytokines. Initiators of this inflammation include increased circulating FFAs, mechanical stress caused by AT expansion, hypoxia, damage-associated molecular proteins released by adipocyte death, gut-derived lipopolysaccharide, etc. 21, 146. NF-κB is considered as a signaling intersection in the consequent signal transduction of all potential initiators above, leading to the recruitment of immune cells and secretion of pro-inflammatory cytokines. These cumulative factors lead to hypercytokinemia and chronic systemic inflammation, conducive to hyper-inflammatory response in COVID-19 patients with obesity 142.

2) Impaired virus clearance and high viral load. Chronic physiological changes, disordered metabolism, and dysregulated immune responses in individuals with obesity probably converge to impaired clearance of coronavirus in the early stage of COVID-19. Compared with patients infected with normal BMI, patients with obesity have higher viral load and reach peak earlier, as evidenced in DIO mice infected with H1N1 87, 145. This impaired elimination and high viral load probably fuel extensive virus-induced direct cytopathic effects in the early stage and the resultant production of proinflammatory cytokines such as IL-6, TNF-α, and IL-1β in infected epithelial cells 136.

3) High possibility of the virus spreading to the lower respiratory tract. Compared with control mice with the same viral titer, more influenza virus antigens were present in the bronchioles and alveolar regions of obese mice, suggesting increased virus transmission to the lower respiratory tract 145. Studies in animal models, especially in mice, have demonstrated that different infection sites of human coronavirus can result in distinct outcomes. Compared with infection mainly distributed in respiratory epithelial cells, simultaneous infection of respiratory epithelial cells and alveolar epithelial cells were more likely to cause direct cytopathic effects and severe lesions 147. SARS-CoV-2 in obesity probably also spreads more viruses to the lower respiratory tract and causes more serious lesions.

4) Hyperglycemia. Impaired pancreatic function and/or hyperglycemia existing in obesity probably are further worsened by SARS-CoV-2 infection which attacks ACE2-positive islet cells 46. The resulting hyperglycemia can cause the glycation of proteins and lipids and generate advanced glycation end products that facilitate the production of reactive oxygen species (ROS) through NF-kB-dependent activation. Together with ROS produced by oxidative phosphorylation of excessive blood glucose, these metabolites activate NLRP3 inflammasomes and subsequent IL-1 system 44, thereby fueling cytokine storm, one of the most prominent findings related to hyperglycemia 51.

5) Disordered adipokines. In addition to acting as an energy-storage depot, AT can also secrete adipokines with an immunomodulatory effect as an endocrine organ. The excessive production of proinflammatory leptin and resistin juxtaposed to insufficient anti-inflammatory adiponectin in obesity partially justifies the imperfect immune responses to SARS-CoV-2 145. High plasma leptin in subjects with obesity, along with CXCL-10 and TNF-α, is a predictor of COVID-19 severity and disease progression. Mechanistic studies have revealed that leptin promotes inflammatory M1 polarization of monocytes through STAT3 and NF-κB signaling pathways and upregulates the secretion of cytokines such as IL-6, resulting in excessive inflammatory responses 148. In LPS-induced acute lung injury, resistin increased the sensitivity of neutrophils to LPS exposure and promoted the formation of neutrophil extracellular trap, production of pro-inflammatory cytokines, and the aggravation of pulmonary edema 149. The process of cytokine storm probably was further amplified by the intricate cross-talk between decreased adiponectin and macrophages. Adiponectin induced the production of IL-10 in macrophages, inhibited NF-κB, and directly regulated the expression of IL-1 receptor-associated kinase M which controlled the response and tolerance of macrophage to proinflammatory stimuli 150, 151. Therefore, the immune system of people with low plasma adiponectin levels tends to overreact to pathogens. The lack of negative feedback anti-inflammatory mechanisms is probably conducive to developing the cytokine storm and the susceptibility to fatal outcomes following SARS-CoV-2 infection.

6) Imbalanced RAS. The overall expression level of ACE2 in people with obesity is higher than in their nonobese counterparts. ACE2 falls off after binding with the spike protein of SARS-CoV-2 and therefore fails to produce Angiotensin (1-7), which is supposed to act on the Mas receptor and being protective in vasodilation, anti-inflammation, anti-oxidation, etc. 152. An estimated higher viral load and a possible greater drop of ACE2 in the obese after infection shift the balance of RAS to the pro-inflammatory axis of ACE/Angiotensin II/Angiotensin type 1 receptor, conducive to worse pulmonary vascular permeability, alveolar epithelial cell apoptosis, pulmonary inflammation, etc. 10.

7) Dysregulated IFN-I response. Instead of eliminating coronavirus, dysregulated, delayed, and persistent IFN-I in obesity can selectively induce the expression of transcription factor IRF1 in a STAT1-dependent manner. This is followed by the activation and transcription of pro-inflammatory cytokine genes, including IL-1β, IL-6, and TNF-α, potentially contributing to the development of cytokine storm 153, 154.

8) Inflammatory macrophages. Macrophages account for 40-60% of immune cells in AT in mice with obesity, while only 10-15% in lean mice, standing out as the most abundant immune cells of concern in obesity 155. In individuals with obesity, lipid droplet accumulation, FFAs, local hypoxia, and lipopolysaccharide all polarize macrophages to the M1 pro-inflammatory phenotype (or more specifically, metabolism-activated macrophage phenotype) 156, secreting enormous inflammatory cytokines including IL-6, IL-1β, and TNF-α, and acting as key mediators of inflammation in AT 155. Coexisting conditions in COVID-19 patients with obesity like dysregulated IFN-I response, delayed T cell response, disordered adipokines, and extensive virus-induced cytopathic effects orchestrate excessive infiltration of macrophages in the lung parenchyma. Coincidentally, severe/critical COVID-19 cases are featured by the enormous highly inflammatory macrophages that in turn bring about excessive production of inflammatory cytokines 110. Subsequent recruitment of T lymphocytes and their consequent crosstalk with macrophages through release of cytokines such as IFN exacerbate the inflammatory responses in a vicious circle and culminate in cytokine storm and disease progression. Speranza et al. supported this by finding that alveolar macrophages drove the inflammatory responses during SARS-CoV-2 infection 157. In addition, the glycosylated Fc terminal of IgG shows a stronger affinity for the FcγRIIIa of macrophages, causing macrophage overactivation. Cytokines such as IL-1β, IL-6, and TNF-α amplify the potential inflammatory storm, undermine the pulmonary endothelial integrity, and thus trigger microvascular thrombosis, facilitating the development of severe COVID-19 158, 159. Obesity-related hyperglycemia probably increases IgG glycosylation and sets the stage for uncontrolled cytokine storm 83, 89. In summary, the question of whether and why the cytokine storm will form in subjects with obesity remains an enigma. Therefore, more studies are needed to clarify the role of the pro-inflammatory events proposed above in SARS-CoV-2 infection.

## The prothrombotic state in patients with obesity confer susceptibility to severe COVID-19 and poor prognosis

Universal studies have consistently found that thrombotic microangiopathy, arterial thrombosis, and venous thrombosis are important causes of COVID-19 progression and increase mortality 160. Post-mortem examination of patients with COVID-19 reveals extensive microthrombosis in the lungs, lower extremities, hands, brain, heart, liver, kidney, etc. Primary microthrombus in pulmonary vessels can explain the sudden progression to acute respiratory distress syndrome with marked pulmonary edema and hypoxemia, possibly, while systemic thrombosis contributes to loss of life through multiple organ dysfunction such as acute liver and kidney failure, neurological insults, cardiomyopathy, and mesenteric ischemia 161-163. The hypercoagulable state in patients with COVID-19 is mainly manifested as hypofibrinolysis and increased thrombin generation 164, 165. COVID-19 is also featured by the increased expressions of circulating markers for endothelial injuries, such as von Willebrand factor, angiopoietin 2, plasminogen activator 1, follistatin, and soluble thrombomodulin, among which the first three markers are particularly high in patients in ICU while the last four markers are positively correlated with mortality 160, 166. Hypoxia, vascular endothelial injury, oxidative stress and other stressors in COVID-19 can cause platelet hyperactivation and apoptosis by affecting the metabolism and function of its mitochondria, facilitating platelet-rich thrombotic microangiopathy and critical disease. Additionally, the binding of activated platelets to neutrophils recruited in pulmonary vessels and the subsequent rolling on the endothelium plays a vital role in initiating immune thrombosis 167. It logically follows that pre-existing risk factors in obesity, including endotheliopathy, platelet hyperactivation, hypercoagulability, and impaired fibrinolysis, probably lead to exacerbated thrombosis and severe COVID-19.

1) Endotheliopathy. Due to long-term exposure to inflammatory stimulation, systemic oxidative stress, hyperlipidemia, and other stimulating factors in obesity, endothelial dysfunction act as a principal determinant of microvascular dysfunction by tilting the vascular equilibrium towards constriction. Meanwhile, blood vessels show decreased antioxidant defense approaches and the loss of antithrombotic properties, owing to increased ROS production, adhesion molecules, and endothelin-1 168. 2) Platelet hyperactivation. The increased platelet size and volume in subjects with obesity represents higher susceptibility to activation, while elevated expression of CD40L and P-selectin in vivo confirms continued activation of platelets and endothelial cells 169. Weight gain also weakens the sensitivity of platelets to insulin, prostacyclin, and nitric oxide and endows platelets a higher prothrombotic response to aggregators 170, 171. The fatal combination of endotheliopathy and platelet hyperactivation in individuals with COVID-19 is highlighted by the positive association between increased ICU admission/death and elevated levels of biomarkers of this alliance 160, 166. 3) Hypercoagulability. Elevated levels of tissue factor, von Willebrand factor, prothrombin, fibrinogen, and factors VII and VIII place patients with obesity in a mild to moderate hypercoagulable state 172. 4) Impaired fibrinolysis. Obesity impairs the fibrinolytic system by significantly upregulating the expression of plasminogen activator inhibitor 1 which inhibits plasminogen activator and limits the dissolution of fibrin clots 173. Together, concomitant events in obesity favor a prothrombotic environment and contribute to thrombosis following a positive COVID-19 diagnosis, possibly.

## Potential therapeutic opportunities for COVID-19 individuals with obesity

Unfortunately, a proverbial “silver bullet” for severe COVID-19 has not been found yet due to its multifaceted causes. A multi-pronged approach is therefore needed to manage the current crisis. In addition to the medicines disrupting viral replication and dissemination, the enormous benefits of modifiable lifestyle like diet and physical training in obesity should not be marginalized. For example, moderate weight loss and physical activity can reshape AT distribution, increase muscle mass, HDL levels and insulin sensitivity, and even augment vitamin D synthesis when outdoor activities are involved.

Host-directed therapies that restrain abnormalities and restore required responses in patients with obesity are also available as alternatives. Good adherence to prior treatment and control of comorbidities is indispensable for people with underlying diseases. It was once suspected that angiotensin receptor blockers/angiotensin-converting enzyme inhibitors probably increased individual susceptibility to COVID-19 and worse outcomes through the upregulation of ACE2. However, discontinuation of these two types of drugs in hypertensive patients is not recommended according to the large-scale retrospective cohort studies that disproved this doubt 174, 175. The contribution of dyslipidemia to COVID-19 susceptibility and severity in patients with obesity opens the possibility that lipid-lowering therapeutics probably ameliorate the consequences brought by high cholesterol in circulation and/or tissues. For example, statins, a class of shelved lipid-lowering medicine drug, was verified to be associated with lower mortality. The 28-day all-cause mortality in the matched non-statin and statin groups was 9.4% and 5.2%, respectively, supporting statins as adjuvant therapy for COVID-19, especially in patients with obesity 176. As for hyperglycemia, improvement in morbidity by well-controlled blood glucose (fluctuating within 3.9-10.0 mmol/L) in patient outcome has been demonstrated by Zhu et al. who has proved that this glycemic variability is associated with lower mortality compared to those whose upper limit of blood glucose exceeded 10.0 mmol/L during hospitalization 177. Therefore, early detection and glycemic control in the clinical management of COVID-19 is crucial. It should be noted that medications like glucocorticoids, severe illness, and infection often necessitate timely and reasonable adjustment of hypoglycemic drugs and/or insulin dosage based on their impairment of insulin sensitivity. In addition, observational studies showed the safety and merits of vitamin D supplementation in acute respiratory infections. The obese suffering from severe vitamin D deficiency possibly benefits the most from supplementation 60. However, randomized clinical trials yielded controversial results 178. Specific recommendations for vitamin D supplementation in COVID-19 patients still need to be clarified, but patients with obesity should be beyond the scope of the debate since correcting their vitamin D deficiency is what they need. Therefore, exposure to sunlight and, if possible, artificial ultraviolet B, and vitamin D supplementation seem to be appealing adjunct therapies for obese patients.

Given the distinct roles of IFN-I in obese patients at different stages of COVID-19, an IFN-based therapeutic schedule should be refined in specific situations. During the acute phase of SARS-CoV-2 infection, prophylactic treatment with IFN-I, which is immuno-stimulatory and anti-viral effective, can confer maximum protection without causing significant systemic inflammation and appreciable pathology. Once delayed and persistent production of IFN-I drives excessive inflammatory responses and progressive tissue damage, the optimal option is to block its pathways downstream. However, the non-inflammatory, long-lasting, and focused expression pattern of IFN-III allows it to be applied through the whole course of treatment, providing lasting anti-viral protection at the entrance and preferential targets of SARS-CoV-2 (the upper respiratory tract and lungs) 87. Notably, IFN-stimulated expression of ACE2 in the target cells of SARS-CoV-2, thus enhancing viral infectivity, has introduced more complexity to the clinical application of IFN in COVID-19 management 179. Further unbiased and strictly controlled studies with granularity are warranted to develop rationale-based interventions and maximize therapeutic effect.

Obtaining the dynamics of cytokines in COVID-19 patients with obesity is pivotal since different degradation rates of various cytokines narrow down the therapeutic window for their respective antagonists. According to the development of cytokine storm responsible for severe COVID-19, strategies directed at attenuating specific processes are beneficial, possibly. This speculation has been supported by the clinical results obtained from the treatment of severe COVID-19 with tocilizumab, a monoclonal antibody targeting IL-6 pathways. Other potential therapeutic choices, including targeted IL-1, IL-2, IL-1β, and TNF-α, also have a promising, and yet to be fully explored, role in clinical treatment. Luckily, the anti-inflammatory profiles of drugs with proven safety can be exploited to address the urgent need. For example, statins show anti-inflammatory effects and association with lower mortality 176; meanwhile, hypoglycemic metformin limits the secretion of inflammatory cytokines by inhibiting ROS production and NF-κB signaling 180. In a retrospective cohort analysis, metformin demonstrated a notable association with lower mortality in women with obesity or type 2 diabetes. This sex-specific anti-inflammatory effect resulted from the greater reduction of IL-6 and TNF-α, was possibly crucial to COVID-19 severity 181. Although it has been argued that this favorable anti-inflammatory influence should be weighed up against the theoretical possibility of over-immunosuppression and consequently delayed virus clearance, such strategies can be particularly relevant for obese patients with pre-existing hyperglycemia and hyperlipidemia. After a long bitter dispute, WHO issued guidelines and suggested the systemic application of corticosteroids in critically unwell patients according to its association with reduced mechanical ventilation and short-term mortality 182. Cai et al. further proposed the criteria for corticosteroid treatment should be defined as neutrophil-to-lymphocyte ratio>6.11 at admission and absence of T2DM 183.

As for the hypercoagulable state of obese patients with COVID-19, low-molecular-weight heparin is recommended for anticoagulation because of its beneficial effects on the cumulative incidence of thromboembolic events and death risk 184. Levi et al. suggested that low-molecular-weight heparin should be prophylactically used in all hospitalized COVID-19 patients in the absence of medical contraindications. However, the evidence for this suggestion is insufficient, and further prospective randomized controlled trials are needed 162. Given the importance of endotheliopathy and thrombocytopathy in the pathophysiological changes in COVID-19, patients with obesity probably benefit from therapies targeting endothelial cell and platelet activation, such as nitric oxide and prostacyclin.

The protracted war against the COVID-19 pandemic begins to dawn with vaccine development and widespread vaccination (2378.48 million vaccine doses have been administered as of 16 June 2021), which are particularly important for people with obesity considering their high risk of severe COVID-19 185, 186. However, lessons learned from influenza vaccination suggest that vaccines may not be a panacea for people with obesity. The obese probably also show reduced efficacy of SARS-CoV-2 vaccine similar to influenza vaccine due to immune senescence 71, 114. Sheridan et al. also found that participants with obesity cannot maintain long-term antibody responses 148. Compared with healthy weight individuals, participants with obesity show a greater decline in influenza antibody titers, CD8+ T cell activation, and expression of functional proteins (IFN-γ and granzyme B) 12 months after influenza vaccination 148. The suboptimal response of people with obesity to the vaccine suggests that a tailored vaccination with increased doses and/or times is necessary, and the possibility of infection with SARS-CoV-2 after vaccination cannot be ruled out given weakened protection of vaccines in people with obesity.

In the context of the obesity epidemic, unraveling the mechanism behind severe COVID-19 in individuals with obesity and a thorough understanding of their interactions are requisites for saving severe cases and reducing the death toll. Attributing any apparent public health success or failure to a single factor is certainly tempting, yet such a simplistic approach to the complexities of pathophysiology is unlikely to provide enough insights against COVID-19. By bridging work done in basic pathophysiology, metabolism, immunity, inflammation, and coagulation, researchers can gain a holistic view of how obesity affects the condition and outcome of COVID-19. Ultimately, lessons learned from COVID-19 in obesity provide, despite its inherent tragedy, interesting and new insights for fighting against possible pandemics in the future.

## Abbreviations

ACE2: angiotensin-converting enzyme receptor 2
AT: adipose tissue
BALF: bronchoalveolar lavage fluid
BMI: body mass index
COVID-19: coronavirus disease 2019
DCs: dendritic cells
DIO: diet-induced obesity
FFAs: free fatty acids
FTO: fat mass and obesity-associated gene
HDL: high density lipoprotein
ICU: intensive care unit
IFN: interferon
IL: interleukin
iNKT: invariant natural killer T
IRF9: interferon regulatory factor 9
ISGs: interferon stimulating genes
LDL: low-density lipoprotein
MC4R: melanocortin 4 receptor
NK: natural killer
PPAR: peroxisome proliferator-activated receptor
RAS: renin-angiotensin system
ROS: reactive oxygen species
SARS-CoV-2: severe acute respiratory syndrome coronavirus 2
STAT: signal transducer and activator of transcription
T2DM: type 2 diabetes
TLRs: toll-like receptors
TNF-α: tumor necrosis factor α
WHO: World Health Organization
